# Structures, dynamics, and hydrogen-bond interactions of antifreeze proteins in TIP4P/Ice water and their dependence on force fields

**DOI:** 10.1371/journal.pone.0198887

**Published:** 2018-06-07

**Authors:** Hwankyu Lee

**Affiliations:** Department of Chemical Engineering, Dankook University, Yongin-si, Gyeonggi-do, South Korea; Yonsei University, REPUBLIC OF KOREA

## Abstract

Tenebrio molitor antifreeze protein (TmAFP) was simulated with growing ice-water interfaces at a realistic melting temperature using TIP4P/Ice water model. To test compatibility of protein force fields (FFs) with TIP4P/Ice water, CHARMM, AMBER, and OPLS FFs were applied. CHARMM and AMBER FFs predict more β-sheet structure and lower diffusivity of TmAFP at the ice-water interface than does OPLS FF, indicating that β-sheet structure is important for the TmAFP-interface binding and antifreeze activity. In particular, CHARMM FF more clearly distinguishes the strengths of hydrogen bonds in the ice-binding and non-ice-binding sites of TmAFP than do other FFs, in agreement with experiments, implying that CHARMM FF can be a reasonable choice to simulate proteins with TIP4P/Ice water. Simulations of mutated TmAFPs show that for the same density of Thr residues, continuous arrangement of Thr with the distance of 0.4~0.6 nm induces the higher extent of antifreeze activity than does intermittent arrangement of Thr with larger distances. These findings suggest the choice of CHARMM FF for AFP-TIP4P/Ice simulations and help explain the relationship between Thr-residue arrangement and antifreeze activity.

## Introduction

Antifreeze proteins (AFPs), which consist of polypeptides with various sizes and structures, are found in arctic or antarctic organisms such as bacteria [1], fungi [2], plants [3], insects [4], and fish [5–7] that can survive at temperatures even lower than -30°C. AFPs have shown great potential for industrial applications such as cryopreservation [8], food processing [9], and hydrate inhibition [10], since they bind to specific planes of the growing ice crystal and inhibit ice growth, showing a noncolligative property [11–13], which can overcome the limitation of synthetic polymer-based antifreezes that typically require high concentrations. To increase the antifreeze efficiency of AFP, the structure of AFP and its interaction with specific ice plane need to be understood, which has motivated many experimental and theoretical studies.

The DeVries group pioneered experimental and theoretical studies on the interactions between AFPs and ice-water interfaces. They found that AFPs adsorb to ice surfaces and inhibit ice growth through a noncolligative process, which was explained by a mechanism of the melting-point depression on the curved ice-water interface, called the “Gibbs-Thomson” (or Kelvin) effect [7, 14, 15]. Graether observed that the mutation of the ice-binding site of AFP significantly reduces antifreeze activity [4], and Davies and coworkers showed the effects of AFP structure and ice-plane type on the AFP-ice binding [16–18], which were interpreted by hydrogen-bond interactions. Meister et al. observed different hydration layers on the ice-binding sites of different AFPs [19, 20], and Olijve et al. showed that the efficiency of AFP cannot be determined by any single factor, but by a combination of ice-plane type, adsorption rate of AFP, surface coverage, and kinetics of ice nucleation [21]. Liu et al. showed that ice nucleation is depressed by the non-ice-binding face (bulky hydrophobic and charged groups) of AFP but promoted by the ice-binding face (hydroxyl groups) of AFP [22]. While these experiments have shown the dependence on the AFP structure and ice-plane type and proposed the mechanism of the Gibbs-Thomson effect, the results are not always easy to interpret at the level of individual molecules or specific hydrogen-bond interactions between AFPs and water molecules because of the limited resolution of experimental techniques.

To support and complement experimental observations, molecular dynamics (MD) simulations have been performed. McDonald et al. simulated a winter flounder AFP (wfAFP) with pyramidal planes, showing the adsorption of wfAFP to ice surface because of the interactions between Threonine (Thr) residues and oxygen atoms in the ice lattice [23]. Cheng and Merz also found that aspartic acid (Asp), asparagine (Asn), and Thr residues of wfAFP form hydrogen bonds with ice surfaces, to an extent dependent on the distance and ordering of those residues [24]. Dalal et al. observed that the extent of hydrogen bond does not significantly differ in the ice-water interface and bulk water, implying that the protein-ice binding is mainly due to van der Waals forces [25]. Wierzbicki et al. calculated free energies and found that wfAFPs do not directly bind to the ice surface but instead interact with the ice-water interface in the semisolid-semiliquid phase [26]. To more realistically simulate the AFP-ice binding, Nada and Furukawa simulated wfAFP with a growing ice-water interface composed of six-site water molecules at the actual melting point of water and showed that wfAFPs become partially surrounded by the grown ice, leading to a decrease in the velocity of ice growth [27], which supports the Gibbs-Thomson effect. They also observed the dependence of the wfAFP-ice binding on the ice plane, in agreement with experiments [11]. Recently, Kuiper et al. simulated a spruce budworm AFP (sbwAFP) with prism planes, showing that sbwAFP binds to ordered water molecules near the prism face, which can be disrupted by replacing Thr with Leu [28]. Chakraborty and Jana found that water molecules near the ice surface are anchored to hydroxyl groups of Thr [29, 30]. These simulations have captured hydrogen-bond interactions between AFPs and ordered water molecules near the curved ice-water interface [31, 32], although the dependence of antifreeze activity on Thr arrangement, which might be also influenced by different force fields (FFs) in simulation studies, has not been systematically studied through computation.

As a further step toward understanding the effect of Thr arrangement on antifreeze activity, we here report all-atom MD simulations of Tenebrio molitor AFP (TmAFP) with ice-growing surfaces at a realistic melting temperature using TIP4P/Ice water model [33]. We first test compatibility of TIP4P/Ice water with different protein FFs such as CHARMM [34, 35], AMRBER [36, 37], and OPLS [38, 39]. Secondary structures, hydrogen-bond lifetimes, and antifreeze activities of TmAFPs are compared to suggest the FF that is most compatible with TIP4P/Ice water. Then, some of Thr residues of TmAFP are mutated and simulated, showing the effect of Thr-residue arrangement on antifreeze activity, which is rationalized by considering hydrogen-bond interactions between Thr residues and ordered water at the ice-water interface. We will show that these results help explain experimental observations regarding the AFP-ice binding strength and antifreeze activity, to an extent that depends on the arrangement of Thr residue.

## Methods

All simulations and analyses were performed using the GROMACS5.1.4 simulation package [40–42]. The structure and coordinates of TmAFP (QCTGG ADCTS CTGAC TGCGN CPNAV TCTNS QHCVK ANTCT GSTDC NTAQT CTNSK DCFEA NTCTD STNCY KATAC TNSSG CP) were downloaded from the Protein Data Bank (PDB code:1EZG) [43], and then the mutated TmAFPs were generated by replacing some of Thr residues with Leu using the Swiss-Pdb viewer (Table 1) [44]. For water and ice, TIP4P/Ice model [33] was used because this model reproduces more realistic melting temperature of ~260 K than do other water models such as TIP3P (146 K) [45], TIP4P (232 K) [45], TIP4P/Ew (245 K) [46], SPC (190 K) [47], and SPC/E (215K) [48]. Note that TIP5P [45] and six-site [49] water models also show realistic melting temperatures, but they are not considered in this work because they are computationally very expensive. For the ice-crystal plane, Drori et al.’s recent experiments showed that TmAFP adsorb to both prism and basal planes, but the adsorption rate is much faster at the prism plane than at the basal plane [50]. Thus, the prism plane was used in this work, and its coordinate was obtained from Kuiper et al.’s work [28]. To test the ability of different FFs to predict the structure of TmAFPs and their interactions with TIP4P/Ice water, TmAFP was modeled with different FFs such as CHARMM [34, 35], AMBER [36, 37], and OPLS FFs [38, 39].

**Table 1:** List of simulations.

Name	Force field	Mutated amino acids	No. of simulations
TmAFP	CHARMM	-	5
	AMBER	-	5
	OPLS	-	5
TmAFP-m1	CHARMM	T26L T38L T50L T62L	3
TmAFP-m2	CHARMM	T16L T38L T40L T62L T64L	3
TmAFP-m3	CHARMM	T16L T26L T28L T50L T52L T62L T64L	3

**Fig 1:**
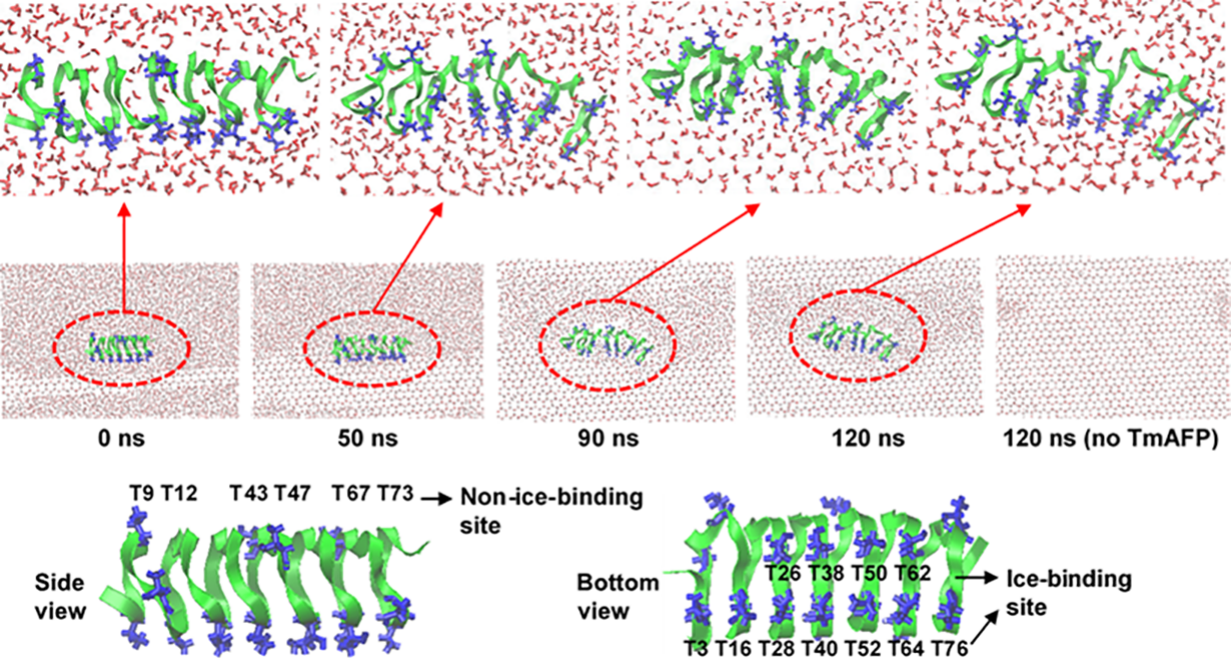
Snapshots from the beginning (0 ns, left) to the end (120 ns, right) for the simulation of TmAFP in water. Green ribbons and blue lines respectively represent the backbone and Thr residues of TmAFP, while oxygen and hydrogen atoms of water are represented as red and white dots, respectively. TmAFP and the surrounding water (or ice) region are magnified (top), and the side and bottom views of TmAFP are depicted (bottom). The images were created using Visual Molecular Dynamics [56].

A single TmAFP was placed above the ice-water interface with a z-directional distance of ~1.8 nm between the protein center and the ice-water interface, and 2 counterions (Na^+^) were added to establish electro-neutrality in a periodic box of size 5.1 × 12.6 × 8.6 nm^3^ (Fig 1). A temperature of 260 K and a pressure of 1 bar were maintained by using a velocity-rescale thermostat [51] and Parrinello-Rahman barostat [52] in the NPT ensemble. A real space cutoff of 1.2 nm was applied for Lennard-Jones and electrostatic forces with the inclusion of particle mesh Ewald for long-range electrostatics [53]. The LINCS algorithm was used to constrain the bond lengths [54, 55]. To obtain more samples, three or five simulations were performed for each system for 120 ns with a time step of 2 fs on computer facilities supported by the National Institute of Supercomputing and Networking/Korea Institute of Science and Technology Information with supercomputing resources including technical support (KSC-2017-C3-061). The last-30 ns trajectories were used for analyses.

## Results and discussion

### Effect of different force fields on the interaction between TmAFP and TIP4P/Ice water

**Fig 2:**
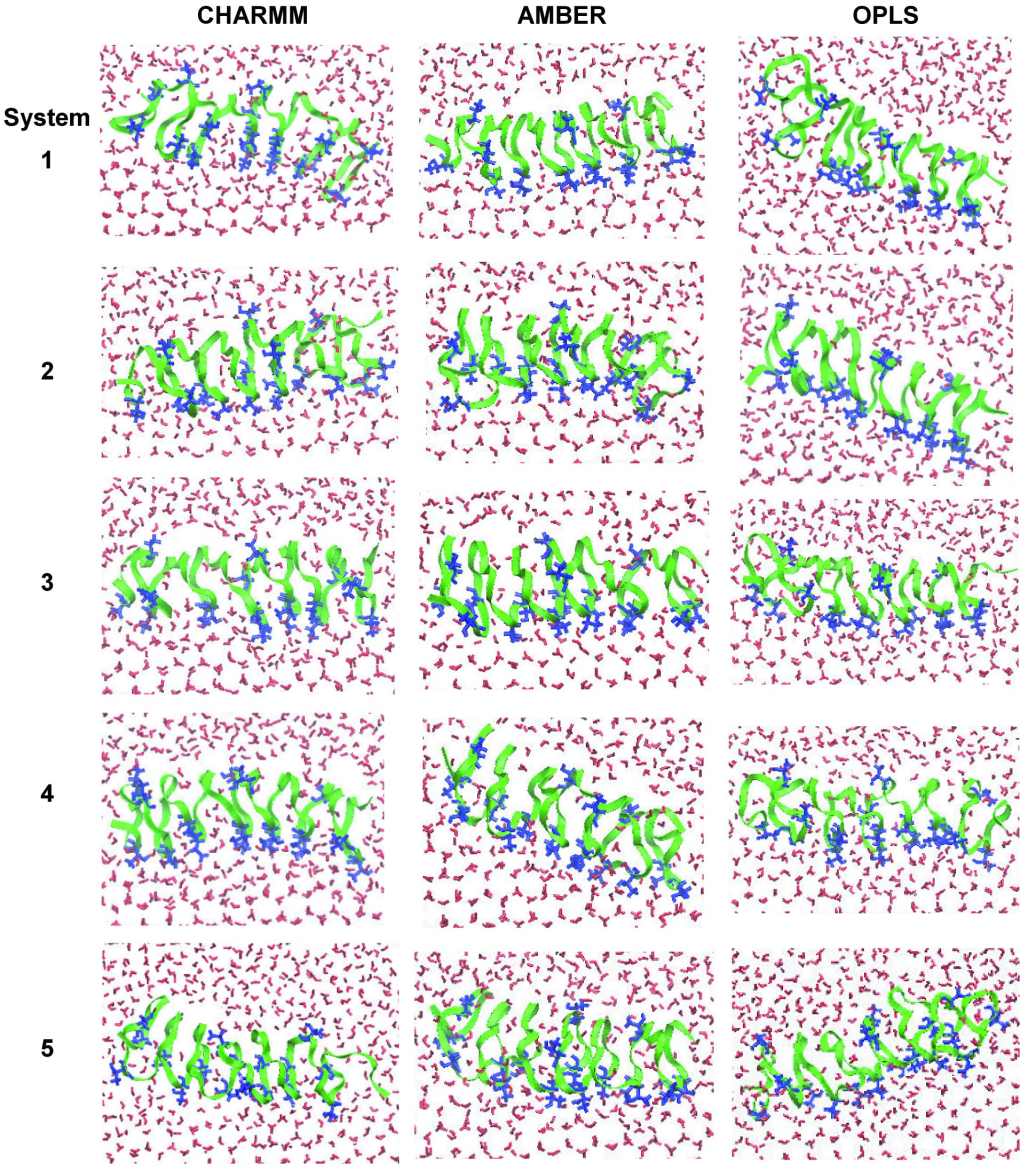
Snapshots at the end of simulations (120 ns) using CHARMM (left), AMBER (middle), and OPLS (right) force fields. Since five simulations were performed for each force field, five snapshots are shown.

Since the TIP4P/Ice water model reproduces the realistic melting temperature of water while still being computationally less expensive than TIP5P or six-site water, this water model has been often used for simulations of AFPs, although the compatibility of TIP4P/Ice water with different protein FFs has not yet been studied. To test the ability of different FFs to predict the strength of hydrogen bonds between AFPs and TIP4P/Ice water, simulations of TmAFP with TIP4P/Ice water were performed using different FFs such as CHARMM, AMBER, and OPLS. Fig 1 shows snapshots for simulations of TmAFP with the growing ice as a function of simulation time. Ice keeps growing until the end of the simulation without TmAFP, while ice growth stops in the presence of TmAFP. Snapshots at 90~120 ns show that the ice-binding site of TmAFP binds to the ordered water of the ice-water interface, leading to the suppression of ice growth. Fig 2 shows snapshots at the end of all simulations using different FFs. Water molecules adjacent to the ice-binding site of TmAFP are highly ordered, indicating the binding between TmAFP and the ice-water interface, which is more significantly observed in CHARMM and AMBER FFs than in OPLS FF. This implies that CHARMM and AMBER FFs can more accurately reproduce the binding of TmAFP with the ice interface and its effect on the suppression of ice growth than does OPLS FF.

**Fig 3:**
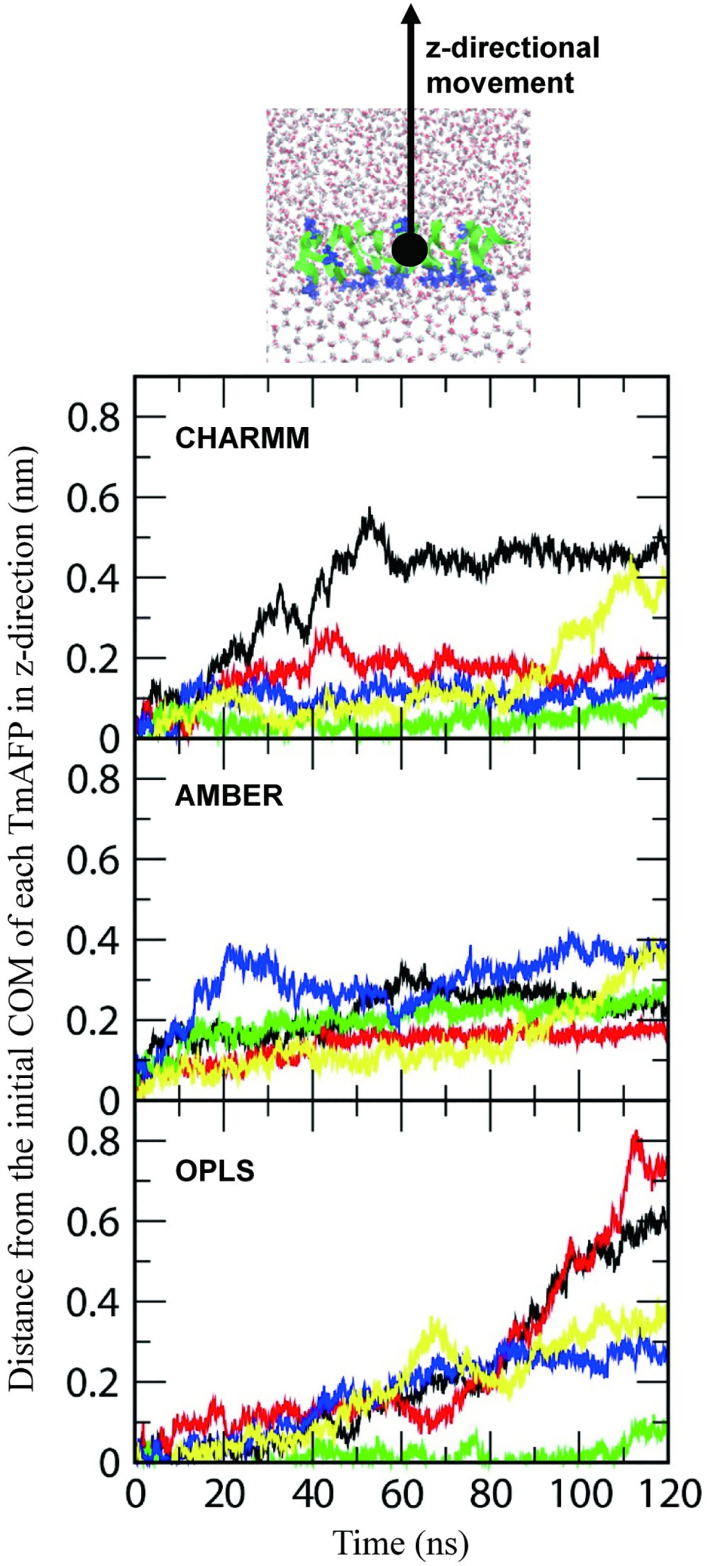
Distances between the center of mass (COM) of TmAFP and its initial COM in the z-direction as a function of time. Since five simulations were performed for each force field, five values are represented with different colors, where black, red, green, blue, and yellow lines respectively correspond to systems 1, 2, 3, 4, and 5 in Fig 2.

To quantify this effect of TmAFP, the z-directional movement of TmAFP was analyzed by calculating the z-directional distance between the center of mass (COM) of TmAFP and its initial COM as a function of time. In Fig 3, although there are some exceptions, simulations with CHARMM and AMBER FFs show that most TmAFPs migrate toward the disordered-water region within a distance of 0.5 nm from its initial position and then do not move much after 90 ns, while simulations with OPLS FF show that some TmAFPs (systems 1 and 2) drastically move until the end, indicating that those TmAFPs do not bind to the ice-water interface and thus keep pushed toward the disordered-water region. Recall from Fig 2 that CHARMM and AMBER FFs show the order water adjacent to the ice-binding site of TmAFP, but OPLS FF does not. These results imply that CHARMM and AMBER FFs can more accurately reproduce the binding of TmAFP with the ice interface and its effect on the suppression of ice growth than does OPLS FF.

**Fig 4:**
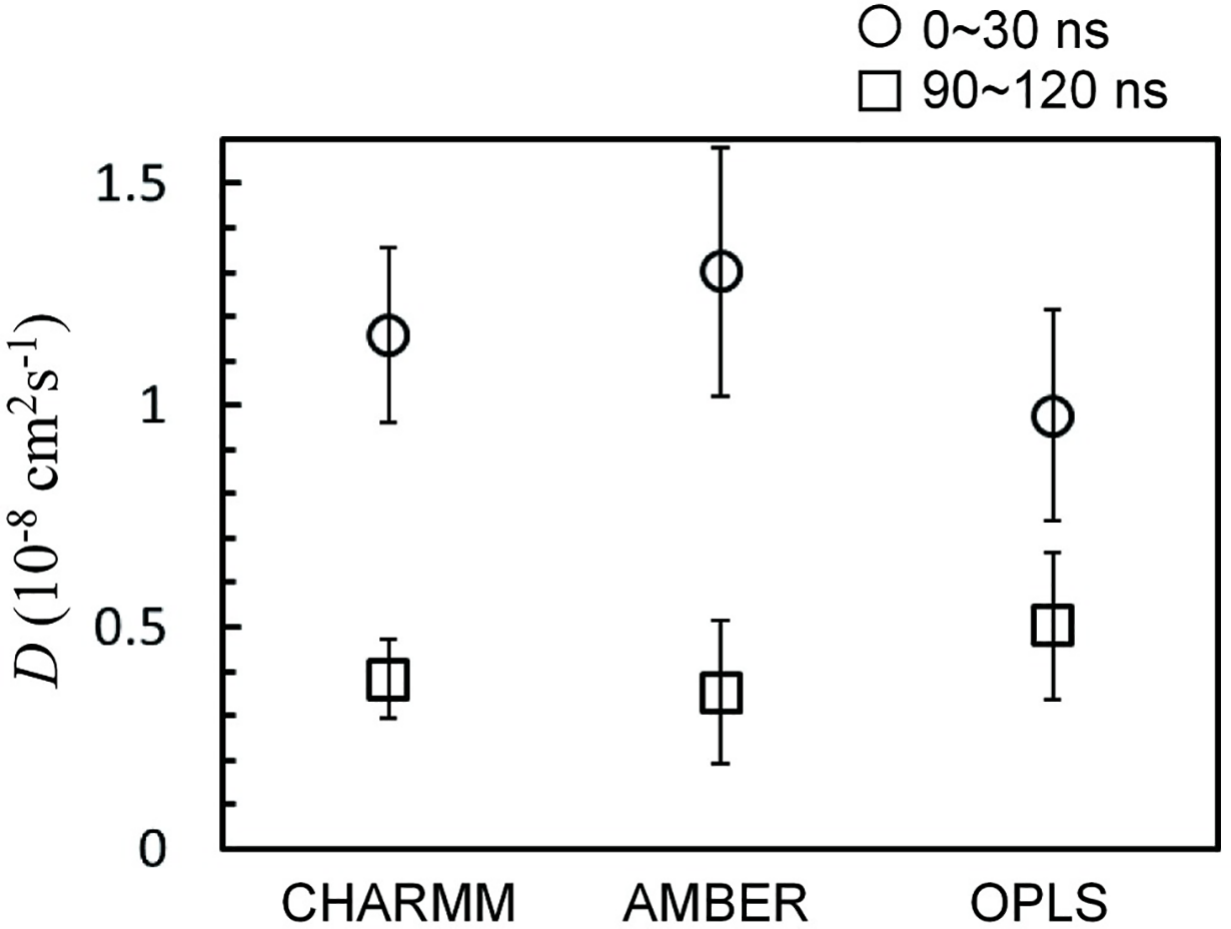
Diffusion coefficients (*D*) of TmAFP at the beginning (0~30 ns) and end (90~120 ns) of simulations. Error bars on points are obtained from standard errors of five *D* values from five simulations.

To confirm these different extents of the TmAFP-ice binding, diffusion coefficients of TmAFPs were calculated by obtaining the slopes of the mean-square displacements (MSD) of the COM of TmAFP for the initial (0~30 ns) and final (90~120 ns) simulation times. Note that the MSD usually needs to be corrected for finite size effects using Yeh and Hummer’s analytic formula for cubic boxes [57], but for systems in non-cubic boxes, the analytic equation of Yeh and Hummer is not necessarily applicable. Also, the size effects should not significantly affect the comparison of diffusivities within the same box geometry, and thus the finite size effect is not corrected here. In Fig 4, diffusivities significantly decrease at the end of simulations with CHARMM and AMBER FFs, apparently because TmAFPs bind to the ice-water interface and inhibit ice growth, as observed in Figs 2 and 3. For simulations with OPLS FF, the diffusivity does not decrease much, showing that TmAFPs keep migrating toward the disordered-water region. This, combined with Figs 2 and 3, indicates that CHARMM and AMBER FFs can more effectively capture the antifreeze activity of TmAFP than does OPLS FF.

To understand this dependence of the antifreeze effect on FFs, secondary structures of TmAFP were calculated using the DSSP program [58]. Table 2 shows that CHARMM and AMBER FFs predict more β-sheet and less random-coil structures than does OPLS FF. Note that the percentage of β-structure is lower than the experimental value of 40% [59], since solvent conditions differ in experiments and our simulations, which precludes a quantitative comparison of secondary structures. Despite this, CHARMM and AMBER FFs predict more β-sheet structure than does OPLS FF and thus more favorably compares with experimental results. These, combined with Figs 2–4, indicate that TmAFP must retain β-sheet structure to suppress ice growth, which can be captured more effectively by CHARMM and AMBER FFs than by OPLS FF.

**Table 2:** Secondary structures (%) of TmAFP.

	Secondary structure (%)		
	CHARMM	AMBER	OPLS
β-sheet	9.9 ± 0.8	6.9 ± 0.5	2.2 ± 0.7
β-bridge	4.4 ± 0.5	5.6 ± 0.3	2.4 ± 0.8
α-helix	0.0 ± 0.0	0.1 ± 0.1	0.1 ± 0.1
Turn	13.5 ± 1.3	12.8 ± 0.8	14.7 ± 1.7
Bend	31.6 ± 1.4	33.3 ± 0.8	34.2 ± 1.3
Coil	40.5 ± 0.6	41.2 ± 0.8	46.5 ± 1.1

### Strength of hydrogen-bond interactions: comparison of different force fields

**Fig 5:**
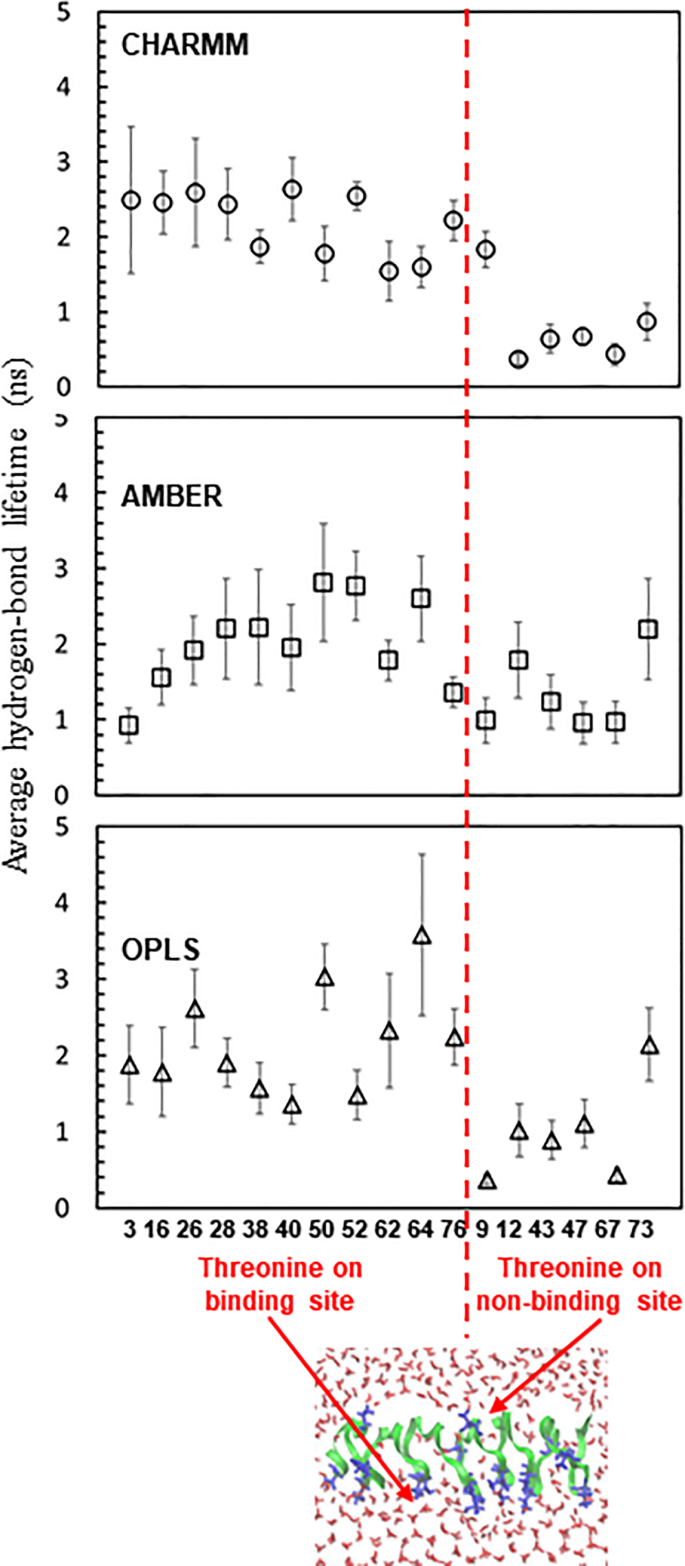
Average lifetimes of hydrogen bonds between Thr residues and water molecules. 11 and 6 Thr residues are respectively classified as the ice-binding and non-ice-binding sites (a red dotted line).

Experimental results have shown that the ice-binding site of AFP consists of many Thr residues and thus forms more hydrogen bonds with the surrounding water than does the non-ice-binding site of AFP [22], implying that the strengths of those hydrogen bonds may also differ. To compare the strengths of hydrogen bonds on the ice-binding and non-ice-binding sites and test the ability of different FFs to predict this, we calculated average lifetimes of hydrogen bonds between Thr residues and water molecules, which is the inverse of the rate constant for hydrogen-bond kinetics (breaking) described by the autocorrelation function of the hydrogen-bonding existence functions (either 0 or 1) averaged over all hydrogen bonds [60, 61]. Here, we assume that a hydrogen-bonding interaction exists when the donor-acceptor distance is less than 0.35 nm and the angle of the hydrogen-donor-acceptor triplet is less than 30° [62]. Analyses were performed using different distance and angle criterions, including 0.3 nm-25° and 0.35 nm-25°, which showed that these stricter criteria produce similar qualitative trends. Thus, the 0.35 nm-30° criterion was used for our hydrogen-bonding analysis of all the simulations. 11 Thr residues, which are regularly distributed along two lines on the bottom side of TmAFP, are considered to be an ice-binding site, while the remaining 6 residues are considered a non-ice-binding site (Fig 1). In Fig 5, simulations with CHARMM FF show that average hydrogen-bond lifetimes are 1.54~2.63 and 0.37~1.83 ns, respectively, for the ice-binding and non-ice-binding sites of TmAFP, indicating the formation of more persistent hydrogen bonds on the ice-binding site than on the non-ice-binding site, in agreement with experiments [22]. The similar trend was also observed for other FFs, but hydrogen-bond lifetimes of the ice-binding and non-ice-binding sites are more clearly distinguished in CHARMM FF than in other FFs. Also, recall that β-sheet structure is more predominantly observed in CHARMM FF than in other FFs (Table 2). These results indicate that when TIP4P/Ice water is used for AFP-ice simulations, CHARMM FF can be a reasonable choice to predict the experimentally observed β-sheet structure of AFPs and their hydrogen-bond interactions with the ice-water interface.

### Effect of the arrangement of Thr residues on antifreeze activity

**Fig 6:**
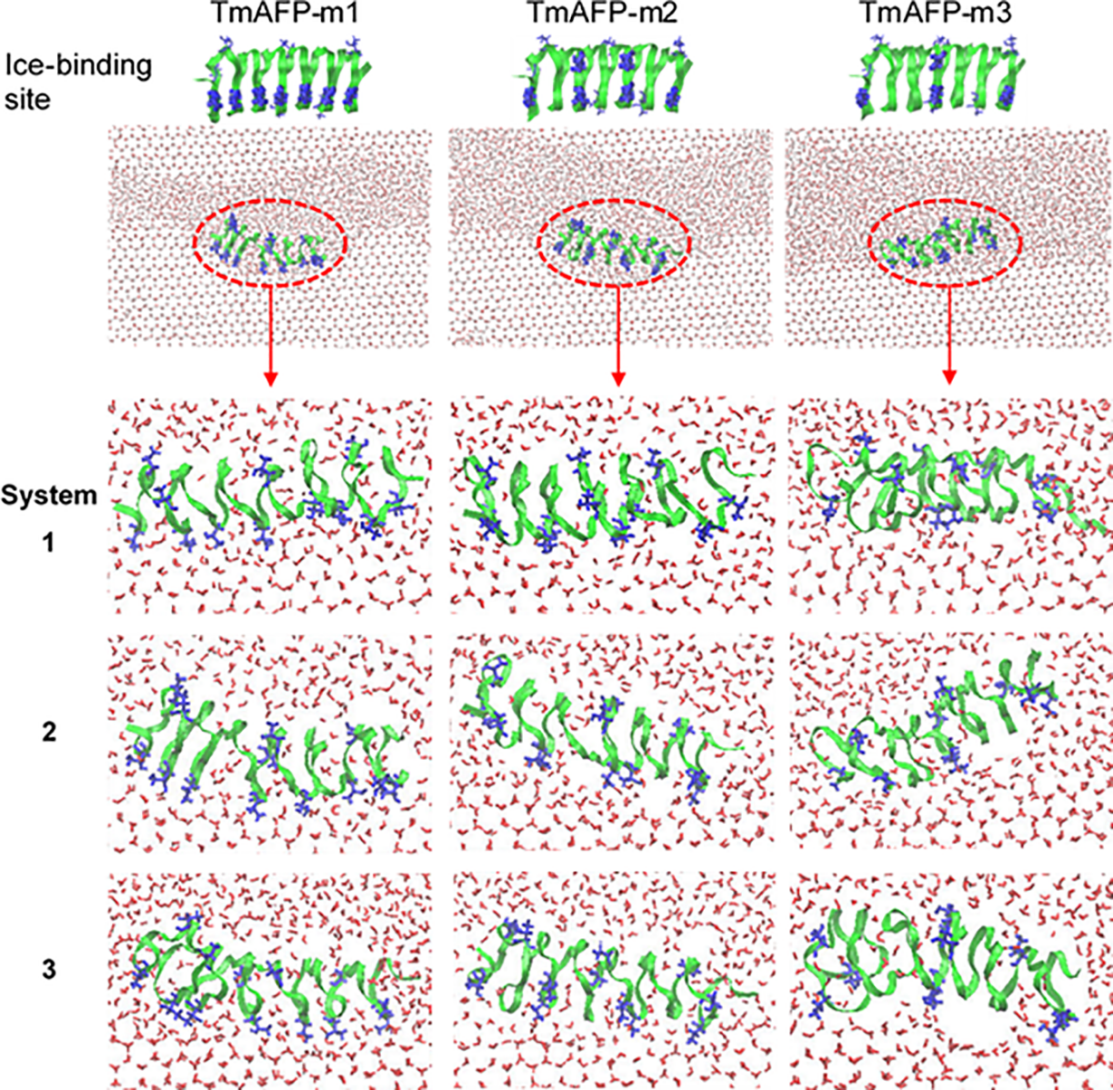
Snapshots at the end (120 ns) of simulations with mutated TmAFP. Since three simulations were performed for each mutated TmAFP, three snapshots are shown (bottom).

**Fig 7:**
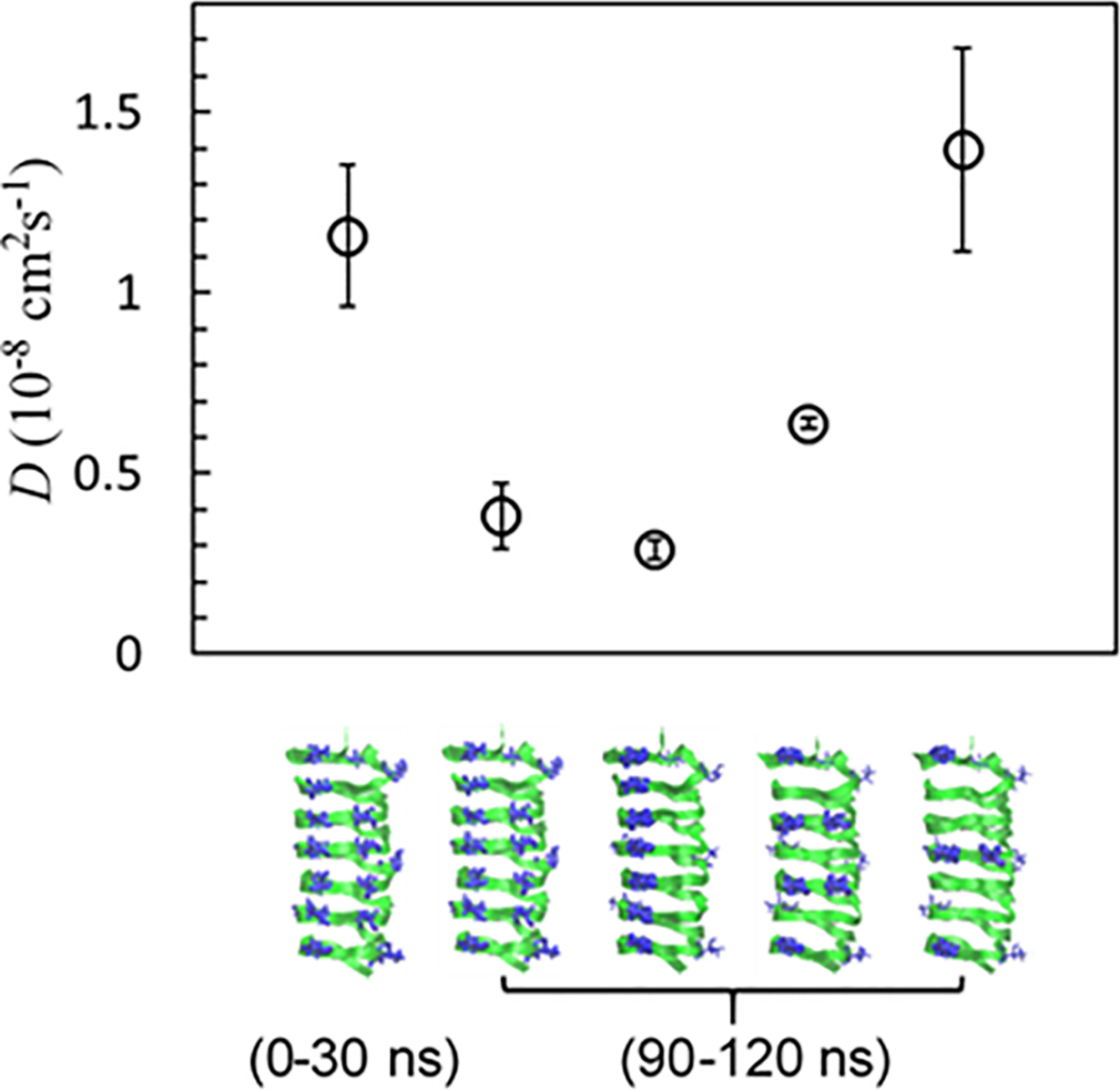
Average diffusion coefficients (*D*) of TmAFP, TmAFP-m1, TmAFP-m2, and TmAFP-m3. Average diffusion coefficients were obtained by averaging those from three or five simulations. Error bars indicate standard errors calculated from the standard deviation of three or five samples (diffusion coefficients) with correction by multiplying a factor of 0.886 or 0.940, respectively [63].

Experimentally, Graether et al. showed that the mutation (Thr to Leu) of the ice-binding site of AFP significantly reduces antifreeze activity [4], which was supported by Kuiper et al.’s simulations [28], indicating that Thr-residue arrangement may influence antifreeze activity. To confirm this, TmAFP was mutated by replacing some of Thr residues with Leu in various patterns and simulated with TIP4P/Ice water using CHARMM FF. Mutations of TmAFP are described in Fig 6 and Table 1, showing that the ice-binding site of TmAFP-m1 has Thr residues along only a single line, and the ice-binding sites of TmAFP-m2 and TmAFP-m3 have intermittent arrangement of Thr residues along two lines. In Fig 6, final snapshots of simulations show that TmAFP-m1 and TmAFP-m2 induce the formation of curved ice-water interfaces, while TmAFP-m3 does not. Fig 6 also shows that the water molecules adjacent to the ice-binding site of proteins are more ordered in TmAFP-m1 than in other mutated ones, indicating the stronger binding of TmAFP-m1 with the ice-water interface. To quantify the extent of antifreeze activity, diffusion coefficients of TmAFP and mutated TmAFPs were compared. Fig 7 shows that diffusivities of TmAFP and TmAFP-m1 become very close at the end of simulations, while diffusivity of TmAFP-m3 at the end of simulation is almost same as that of TmAFP at the beginning of simulation. This indicates that TmAFP-m1 strongly binds to the ice-water interface and thus does not diffuse much, similar to TmAFP, while TmAFP-m3 does not bind to the ice-water interface and thus keeps pushed toward the water region, leading to a significant decrease in antifreeze activity. For TmAFP-m2, diffusivity is higher than TmAFP-m1 and lower than TmAFP-m3. These indicate that the extent of the TmAFP-ice binding strength and antifreeze activity is higher in the order of TmAFP and TmAFP-m1 > TmAFP-m2 > TmAFP-m3, indicating that antifreeze activity can be reduced by replacing Thr with Leu, as observed in previous experiments [4] and simulations [28].

### The relationship between Thr position and the TmAFP-ice binding

**Fig 8:**
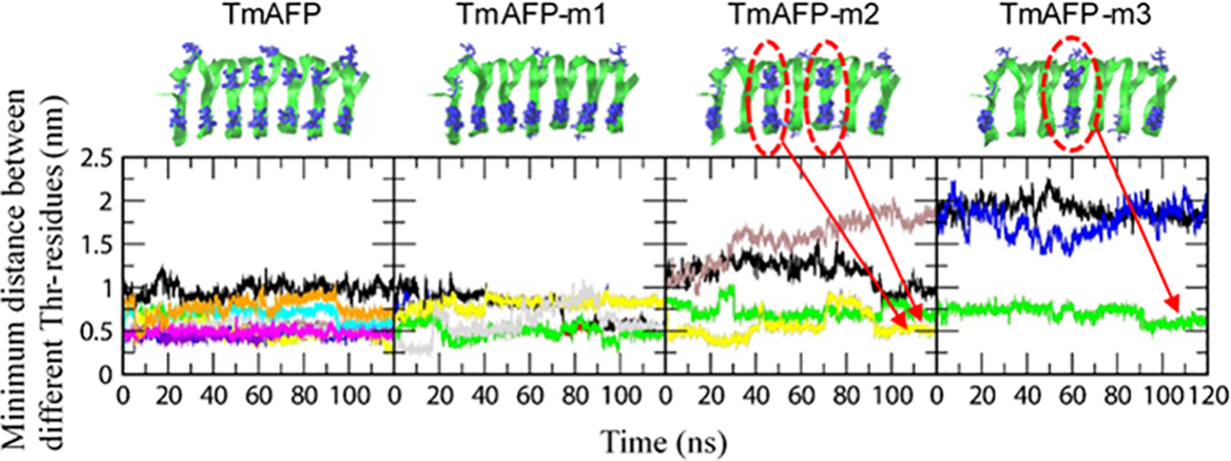
Minimum distances between hydroxyl groups of different Thr residues as a function of time. Since the binding sites of TmAFP, TmAFP-m1, TmAFP-m2, and TmAFP-m3 respectively include 11, 7, 6, and 4 Thr residues, the minimum distance for each Thr residue is represented by different colors. Note that the neighboring residues should have the same minimum distance, and thus their values are often overlapped, as highlighted in TmAFP-m2 and TmAFP-m3.

**Fig 9:**
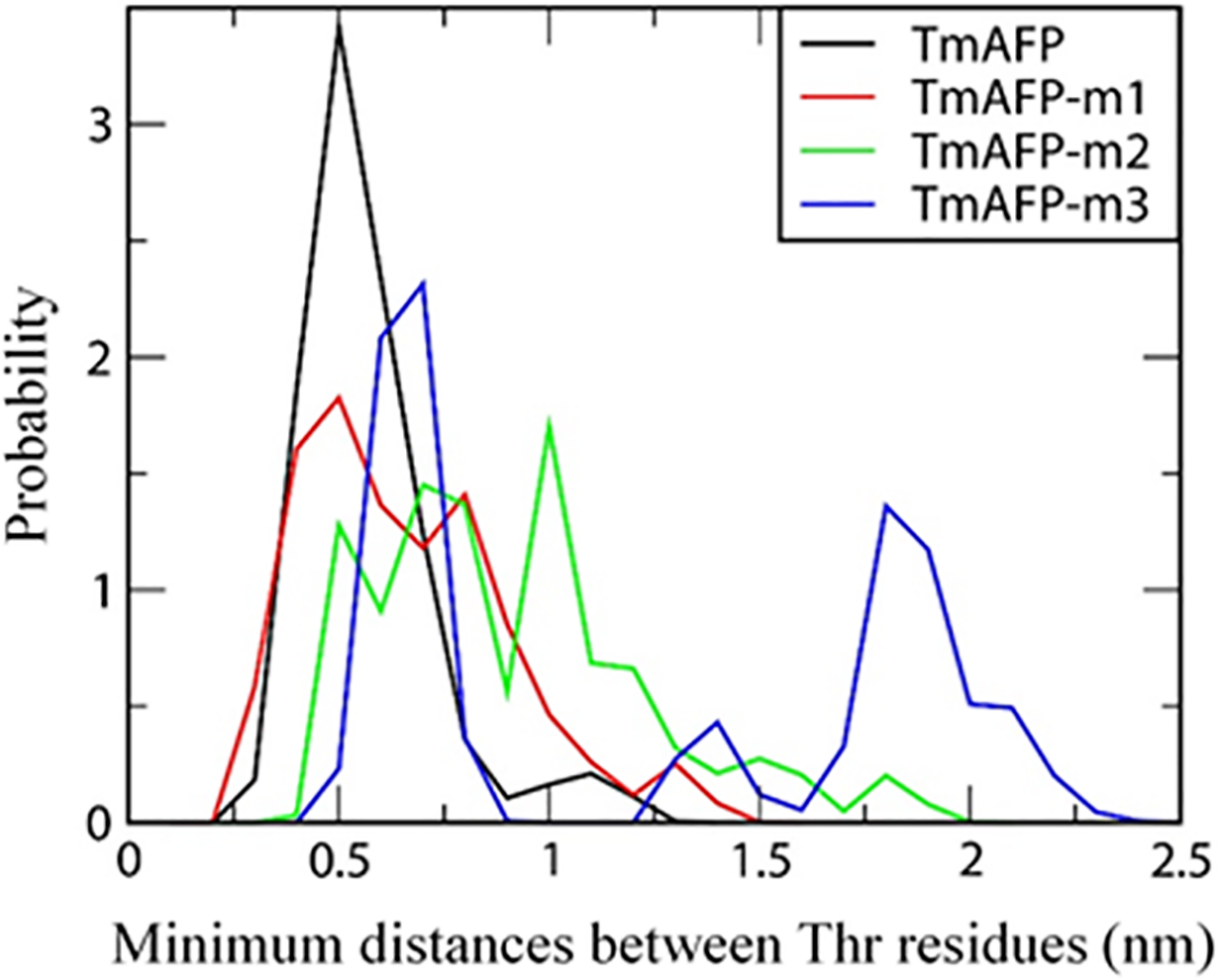
Distributions of minimum distances between hydroxyl groups of different Thr residues for all three or five simulations.

**Fig 10:**
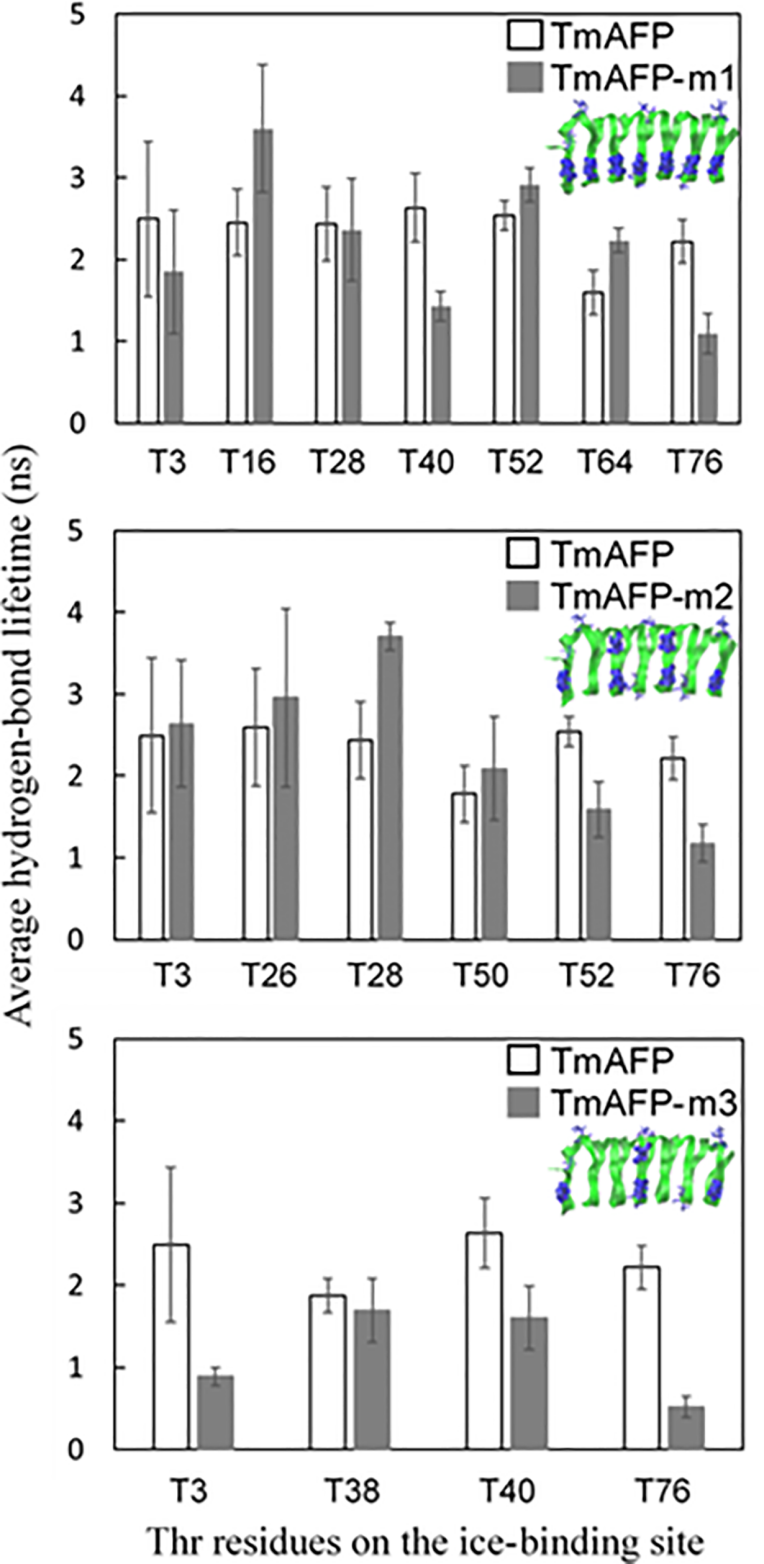
Average lifetimes of hydrogen bonds between ice-water interfaces and Thr residues on the ice-binding sites of TmAFP, TmAFP-m1, TmAFP-m2, and TmAFP-m3.

As discussed above, antifreeze activity of TmAFP is influenced by the arrangement of Thr residues. Since Thr residues of the ice-binding site form strong hydrogen bonds with the ice-water interface and induce antifreeze activity, the strength of the AFP-ice binding may depend on the arrangement of Thr residues, although their relationship has not been systematically studied. To resolve this, we calculated minimum distances between Thr residues. In Fig 8, TmAFP and TmAFP-m1 show that distances between Thr residues are less than 0.9 nm, while TmAFP-m2 and TmAFP-m3 show larger distances. Fig 9 plots the distributions of minimum distances between Thr residues for all simulated systems, showing that minimum distances for TmAFP and TmAFP-m1 are mostly distributed in the range of 0.4~0.6 nm, while those for TmAFP-m2 and TmAFP-m3 are in the broad range of 0.5~2 nm. Recall from Fig 7 that the extent of antifreeze activity is higher in the order of TmAFP ≈ TmAFP-m1 > TmAFP-m2 > TmAFP-m3, indicating that antifreeze activity increases with decreasing the distance between Thr residues. Also, note that the distance between ordered water molecules of ice surface is ~0.45 nm, which is in the minimum-distance range of 0.4~0.6 nm for TmAFP and TmAFP-m1, implying that Thr residues need to be densely spaced for the formation of strong hydrogen bonds. To check this, we calculated average lifetimes of hydrogen bonds between water molecules and Thr residues on the ice-binding sites of mutated TmAFPs. Fig 10 shows more significantly decreased hydrogen-bond lifetimes for TmAFP-m3 than for other mutated ones, indicating weaker hydrogen-bond interaction of TmAFP-m3. These results, combined with Figs 8 and 9, indicate that the replacement of Thr with Leu reduces antifreeze activity of TmAFP, as previously observed in Kuiper et al.’s simulations [28]. In particular, our simulations show that although the same number of Thr residues are distributed on the ice-binding sites of TmAFP-m1 and TmAFP-m2, the extent of antifreeze activity is higher for TmAFP-m1 than for TmAFP-m2. This indicates that the densely distributed arrangement of Thr with the distance of 0.4~0.6 nm more effectively induces antifreeze activity than does the intermittent arrangement of Thr with larger distances, implying the dependence on the distance between Thr residues.

These simulations show the effect of different FFs on the secondary structure and antifreeze activity of TmAFP, and the strength of the TmAFP-ice surface binding, suggesting a choice of CHARMM FF especially for simulations of AFP and TIP4P/Ice water at a realistic melting temperature. In particular, simulations show the higher extent of antifreeze activity when Thr residues are more densely distributed with the distance of 0.4~0.6 nm, indicating the effect of Thr-residue arrangement on hydrogen-bond interactions and antifreeze activity. Note that different types of AFPs have specific binding affinities with various ice-plane types, so our results should be considered for specific AFP and ice plane with caution. To more precisely determine the antifreeze activity of different TmAFPs, it would obviously be interesting to calculate the free energy of binding at the AFP-ice interface, which we hope to report on elsewhere.

## Conclusions

We performed MD simulations of TmAFP with growing ice-water interfaces using TIP4P/Ice water that can reproduce a realistic melting temperature while still being computationally less expensive than TIP5P or six-site water. Since the interactions between proteins and TIP4P/Ice water have not been verified, we first tested compatibility of TIP4P/Ice water with different FFs such as CHARMM, AMBER, and OPLS. TmAFP binds to the ice-water interface, induces curvature of ice surface, and suppresses ice growth, although their extents differ. CHARMM and AMBER FFs predict more β-sheet structure and lower diffusivity of TmAFP at the ice-water interface than does OPLS FF, indicating the stronger TmAFP-ice surface binding and antifreeze activity. In particular, analyses of hydrogen-bond lifetime show that CHARMM FF more clearly distinguishes the strengths of hydrogen bonds in the ice-binding and non-ice-binding sites of TmAFP than do other FFs, which favorably compares with experiments, implying that CHARMM FF can be reasonably chosen to reproduce experimental observations on the secondary structure of AFP and its interaction with TIP4P/Ice water.

To understand the relationship between Thr-residue arrangement and antifreeze activity of AFP, mutated TmAFPs were generated by replacing some of Thr residues with Leu and simulated with TIP4P/Ice water using CHARMM FF. Simulations of mutated TmAFP show that when densities of Thr are same, densely distributed arrangement of the distance of 0.4~0.6 nm more effectively induces antifreeze activity than does intermittent arrangement of Thr with larger distances. This indicates that distances between Thr residues must be small enough to maintain ordered structure of ice-surface water that can form persistent hydrogen bonds with TmAFP. These findings suggest that CHARMM FF can be reasonably chosen for simulations of AFP with TIP4P/Ice water at a realistic melting temperature, as well as help explain experimental observations regarding the difference of the binding strength in the ice-binding and non-ice-binding sites of TmAFP, and the dependence of antifreeze activity on the distance between Thr residues and hydrogen-bond interactions with ice surfaces.

## References

[1] Gilbert JA, Davies PL, Laybourn-Parry J. A hyperactive, Ca2+-dependent antifreeze protein in an Antarctic bacterium. FEMS Microbiology Letters. 2005;245(1):67–72. doi: 10.1016/j.femsle.2005.02.022 15796981 10.1016/j.femsle.2005.02.022

[2] Duman JG, Olsen TM. Thermal hysteresis protein activity in bacteria, fungi, and phylogenetically diverse plants. Cryobiology. 1993;30(3):322–8.

[3] Sidebottom C, Buckley S, Pudney P, Twigg S, Jarman C, Holt C, et al Heat-stable antifreeze protein from grass. Nature. 2000;406(6793):256 doi: 10.1038/35018639 10917518 10.1038/35018639

[4] Graether SP, Kuiper MJ, Gagné SM, Walker VK, Jia Z, Sykes BD, et al β-Helix structure and ice-binding properties of a hyperactive antifreeze protein from an insect. Nature. 2000;406:325 doi: 10.1038/35018610 10917537 10.1038/35018610

[5] DeVries AL, Wohlschlag DE. Freezing Resistance in Some Antarctic Fishes. Science. 1969;163(3871):1073–5. 5764871 10.1126/science.163.3871.1073

[6] Feeney RE, Hofmann R. Depression of freezing point by glycoproteins from an Antarctic fish. Nature. 1973;243(5406):357–9.

[7] Knight CA, DeVries AL. Melting inhibition and superheating of ice by an antifreeze glycopeptide. Science. 1989;245(4917):505–7. doi: 10.1126/science.245.4917.505 17750260 10.1126/science.245.4917.505

[8] Carpenter JF, Hansen TN. Antifreeze protein modulates cell survival during cryopreservation: mediation through influence on ice crystal growth. Proceedings of the National Academy of Sciences. 1992;89(19):8953–7. 10.1073/pnas.89.19.8953 PMC50042 1409591

[9] Ustun NS, Turhan S. Antifreeze Proteins: Characteristics, Function, Mechanism of Action, Sources and Application to Foods. Journal of Food Processing and Preservation. 2015;39(6):3189–97.

[10] Gordienko R, Ohno H, Singh VK, Jia Z, Ripmeester JA, Walker VK. Towards a Green Hydrate Inhibitor: Imaging Antifreeze Proteins on Clathrates. PLOS ONE. 2010;5(2):e8953 doi: 10.1371/journal.pone.0008953 20161789 10.1371/journal.pone.0008953 PMC2820087

[11] Yeh Y, Feeney RE. Antifreeze proteins: Structures and mechanisms of function. Chemical Reviews. 1996;96(2):X–617. 10.1021/cr950260c 11848766

[12] Hew CL, Yang DSC. Protein interaction with ice. European Journal of Biochemistry. 1992;203(1–2):33–42. 1730239 10.1111/j.1432-1033.1992.tb19824.x

[13] Barrett J. Thermal hysteresis proteins. International Journal of Biochemistry and Cell Biology. 2001;33(2):105–17. 11240367 10.1016/s1357-2725(00)00083-2

[14] Raymond JA, DeVries AL. Adsorption inhibition as a mechanism of freezing resistance in polar fishes. Proceedings of the National Academy of Sciences of the United States of America. 1977;74(6):2589–93. 267952 10.1073/pnas.74.6.2589 PMC432219

[15] Knight CA, Cheng CC, DeVries AL. Adsorption of alpha-helical antifreeze peptides on specific ice crystal surface planes. Biophysical Journal. 1991;59(2):409–18. doi: 10.1016/S0006-3495(91)82234-2 2009357 10.1016/S0006-3495(91)82234-2 PMC1281157

[16] Davies PL, Baardsnes J, Kuiper MJ, Walker VK, Hall D, Marahiel MA, et al Structure and function of antifreeze proteins. Philosophical Transactions of the Royal Society B: Biological Sciences. 2002;357(1423):927–35. doi: 10.1098/rstb.2002.1081 12171656 10.1098/rstb.2002.1081 PMC1692999

[17] Davies PL, Hew CL. Biochemistry of fish antifreeze proteins. FASEB Journal. 1990;4(8):2460–8. 2185972 10.1096/fasebj.4.8.2185972

[18] Pertaya N, Marshall CB, Celik Y, Davies PL, Braslavsky I. Direct visualization of spruce budworm antifreeze protein interacting with ice crystals: Basal plane affinity confers hyperactivity. Biophysical Journal. 2008;95(1):333–41. doi: 10.1529/biophysj.107.125328 18339740 10.1529/biophysj.107.125328 PMC2426666

[19] Meister K, Lotze S, Olijve LLC, Devries AL, Duman JG, Voets IK, et al Investigation of the ice-binding site of an insect antifreeze protein using sum-frequency generation spectroscopy. Journal of Physical Chemistry Letters. 2015;6(7):1162–7. doi: 10.1021/acs.jpclett.5b00281 26262966 10.1021/acs.jpclett.5b00281

[20] Meister K, Strazdaite S, DeVries AL, Lotze S, Olijve LLC, Voets IK, et al Observation of ice-like water layers at an aqueous protein surface. Proceedings of the National Academy of Sciences of the United States of America. 2014;111(50):17732–6. doi: 10.1073/pnas.1414188111 25468976 10.1073/pnas.1414188111 PMC4273357

[21] Olijve LLC, Meister K, DeVries AL, Duman JG, Guo S, Bakker HJ, et al Blocking rapid ice crystal growth through nonbasal plane adsorption of antifreeze proteins. Proceedings of the National Academy of Sciences of the United States of America. 2016;113(14):3740–5. doi: 10.1073/pnas.1524109113 26936953 10.1073/pnas.1524109113 PMC4833260

[22] Liu K, Wang C, Ma J, Shi G, Yao X, Fang H, et al Janus effect of antifreeze proteins on ice nucleation. Proceedings of the National Academy of Sciences of the United States of America. 2016;113(51):14739–44. doi: 10.1073/pnas.1614379114 27930318 10.1073/pnas.1614379114 PMC5187720

[23] McDonald SM, White A, Clancy P, Brady JW. Binding of an antifreeze polypeptide to an ice/water interface via computer simulation. AIChE Journal. 1995;41(4):959–73.

[24] Cheng A, Merz KM Jr. Ice-binding mechanism of winter flounder antifreeze proteins. Biophysical Journal. 1997;73(6):2851–73. doi: 10.1016/S0006-3495(97)78315-2 9414201 10.1016/S0006-3495(97)78315-2 PMC1181192

[25] Dalal P, Knickelbein J, Haymet ADJ, Sönnichsen FD, Madura JD. Hydrogen bond analysis of Type 1 antifreeze protein in water and the ice/water interface. PhysChemComm. 2001;4:1–5.

[26] Wierzbicki A, Dalal P, Cheatham TE Iii, Knickelbein JE, Haymet ADJ, Madura JD. Antifreeze proteins at the ice/water interface: Three calculated discriminating properties for orientation of type I proteins. Biophysical Journal. 2007;93(5):1442–51. doi: 10.1529/biophysj.107.105189 17526572 10.1529/biophysj.107.105189 PMC1948032

[27] Nada H, Furukawa Y. Growth inhibition mechanism of an ice-water interface by a mutant of winter flounder antifreeze protein: A molecular dynamics study. Journal of Physical Chemistry B. 2008;112(23):7111–9. 10.1021/jp711977g 18476736

[28] Kuiper MJ, Morton CJ, Abraham SE, Gray-Weale A. The biological function of an insect antifreeze protein simulated by molecular dynamics. eLife. 2015;4(5). 10.7554/eLife.05142 PMC4442126 25951514

[29] Chakraborty S, Jana B. Molecular Insight into the Adsorption of Spruce Budworm Antifreeze Protein to an Ice Surface: A Clathrate-Mediated Recognition Mechanism. Langmuir. 2017;33(28):7202–14. doi: 10.1021/acs.langmuir.7b01733 28650167 10.1021/acs.langmuir.7b01733

[30] Midya US, Bandyopadhyay S. Interfacial Water Arrangement in the Ice-Bound State of an Antifreeze Protein: A Molecular Dynamics Simulation Study. Langmuir. 2017;33(22):5499–510. doi: 10.1021/acs.langmuir.7b01206 28505449 10.1021/acs.langmuir.7b01206

[31] Kar RK, Bhunia A. Biophysical and biochemical aspects of antifreeze proteins: Using computational tools to extract atomistic information. Progress in Biophysics and Molecular Biology. 2015;119(2):194–204. doi: 10.1016/j.pbiomolbio.2015.09.001 26362837 10.1016/j.pbiomolbio.2015.09.001

[32] Nada H, Furukawa Y. Antifreeze proteins: Computer simulation studies on the mechanism of ice growth inhibition. Polymer Journal. 2012;44(7):690–8.

[33] Abascal JLF, Sanz E, Fernández RG, Vega C. A potential model for the study of ices and amorphous water: TIP4P/Ice. Journal of Chemical Physics. 2005;122(23). 10.1063/1.1931662 16008466

[34] MacKerell AD Jr, Bashford D, Bellott M, Dunbrack RL Jr, Evanseck JD, Field MJ, et al All-atom empirical potential for molecular modeling and dynamics studies of proteins. Journal of Physical Chemistry B. 1998;102(18):3586–616. 10.1021/jp973084f 24889800

[35] Mackerell AD Jr, Feig M, Brooks CL Iii. Extending the treatment of backbone energetics in protein force fields: Limitations of gas-phase quantum mechanics in reproducing protein conformational distributions in molecular dynamics simulation. Journal of Computational Chemistry. 2004;25(11):1400–15. doi: 10.1002/jcc.20065 15185334 10.1002/jcc.20065

[36] Wang J, Cieplak P, Kollman PA. How Well Does a Restrained Electrostatic Potential (RESP) Model Perform in Calculating Conformational Energies of Organic and Biological Molecules? Journal of Computational Chemistry. 2000;21(12):1049–74.

[37] Lindorff-Larsen K, Piana S, Palmo K, Maragakis P, Klepeis JL, Dror RO, et al Improved side-chain torsion potentials for the Amber ff99SB protein force field. Proteins: Structure, Function and Bioinformatics. 2010;78(8):1950–8. 10.1002/prot.22711 PMC2970904 20408171

[38] Jorgensen WL, Maxwell DS, Tirado-Rives J. Development and testing of the OPLS all-atom force field on conformational energetics and properties of organic liquids. Journal of the American Chemical Society. 1996;118(45):11225–36.

[39] Kaminski GA, Friesner RA, Tirado-Rives J, Jorgensen WL. Evaluation and reparametrization of the OPLS-AA force field for proteins via comparison with accurate quantum chemical calculations on peptides. Journal of Physical Chemistry B. 2001;105(28):6474–87.

[40] Hess B, Kutzner C, van der Spoel D, Lindahl E. GROMACS 4: Algorithms for highly efficient, load-balanced, and scalable molecular simulation. Journal of Chemical Theory and Computation. 2008;4(3):435–47. doi: 10.1021/ct700301q 26620784 10.1021/ct700301q

[41] Lindahl E, Hess B, van der Spoel D. GROMACS 3.0: a package for molecular simulation and trajectory analysis. Journal of Molecular Modeling. 2001;7(8):306–17.

[42] Van Der Spoel D, Lindahl E, Hess B, Groenhof G, Mark AE, Berendsen HJC. GROMACS: Fast, flexible, and free. Journal of Computational Chemistry. 2005;26(16):1701–18. doi: 10.1002/jcc.20291 16211538 10.1002/jcc.20291

[43] Liou YC, Tocilj A, Davies PL, Jia Z. Mimicry of ice structure by surface hydroxyls and water of a β-helix antifreeze protein. Nature. 2000;406(6793):322–4. doi: 10.1038/35018604 10917536 10.1038/35018604

[44] Guex N, Peitsch MC. SWISS-MODEL and the Swiss-PdbViewer: An environment for comparative protein modeling. Electrophoresis. 1997;18(15):2714–23. doi: 10.1002/elps.1150181505 9504803 10.1002/elps.1150181505

[45] Mahoney MW, Jorgensen WL. A five-site model for liquid water and the reproduction of the density anomaly by rigid, nonpolarizable potential functions. Journal of Chemical Physics. 2000;112(20):8910–22.

[46] Horn HW, Swope WC, Pitera JW, Madura JD, Dick TJ, Hura GL, et al Development of an improved four-site water model for biomolecular simulations: TIP4P-Ew. Journal of Chemical Physics. 2004;120(20):9665–78. doi: 10.1063/1.1683075 15267980 10.1063/1.1683075

[47] Berendsen HJC, Postma JPM, van Gunsteren WF, Hermans J. Intermolecular Forces. Pullman B, editor: D. Reidel Publishing; 1981.

[48] Kusalik PG, Svishchev IM. The spatial structure in liquid water. Science. 1994;265(5176):1219–21. doi: 10.1126/science.265.5176.1219 17787590 10.1126/science.265.5176.1219

[49] Nada H, Van Der Eerden JPJM. An intermolecular potential model for the simulation of ice and water near the melting point: A six-site model of H2O. Journal of Chemical Physics. 2003;118(16):7401–13.

[50] Drori R, Celik Y, Davies PL, Braslavsky I. Ice-binding proteins that accumulate on different ice crystal planes produce distinct thermal hysteresis dynamics. J R Soc Interface. 2014;11(98). 10.1098/rsif.2014.0526 PMC4233703 25008081

[51] Bussi G, Donadio D, Parrinello M. Canonical sampling through velocity rescaling. Journal of Chemical Physics. 2007;126(1):014101–. doi: 10.1063/1.2408420 17212484 10.1063/1.2408420

[52] Parrinello M, Rahman A. Polymorphic transitions in single crystals: A new molecular dynamics method. Journal of Applied Physics. 1981;52(12):7182–90.

[53] Essmann U, Perera L, Berkowitz ML, Darden T, Lee H, Pedersen LG. A Smooth Particle Mesh Ewald Method. Journal of Chemical Physics. 1995;103(19):8577–93.

[54] Hess B. P-LINCS: A parallel linear constraint solver for molecular simulation. Journal of Chemical Theory and Computation. 2008;4(1):116–22. doi: 10.1021/ct700200b 26619985 10.1021/ct700200b

[55] Hess B, Bekker H, Berendsen HJC, Fraaije JGEM. LINCS: A Linear Constraint Solver for molecular simulations. Journal of Computational Chemistry. 1997;18(12):1463–72.

[56] Humphrey W, Dalke A, Schulten K. VMD: Visual molecular dynamics. Journal of Molecular Graphics. 1996;14(1):33–8. 8744570 10.1016/0263-7855(96)00018-5

[57] Yeh IC, Hummer G. System-size dependence of diffusion coefficients and viscosities from molecular dynamics simulations with periodic boundary conditions. Journal of Physical Chemistry B. 2004;108(40):15873–9.

[58] Kabsch W, Sander C. Dictionary of protein secondary structure: pattern recognition of hydrogen-bonded and geometrical features. Biopolymers—Peptide Science Section. 1983;22(12):2577–637. 10.1002/bip.360221211 6667333

[59] Liou YC, Daley ME, Graham LA, Kay CM, Walker VK, Sykes BD, et al Folding and structural characterization of highly disulfide-bonded beetle antifreeze protein produced in bacteria. Protein Expression and Purification. 2000;19(1):148–57. doi: 10.1006/prep.2000.1219 10833402 10.1006/prep.2000.1219

[60] Luzar A. Resolving the hydrogen bond dynamics conundrum. The Journal of Chemical Physics. 2000;113(23):10663–75.

[61] Luzar A, Chandler D. Hydrogen-bond kinetics in liquid water. Nature. 1996;379(6560):55–7.

[62] Jeffrey GA, Saenger W. Hydrogen Bonding in Biological Structures. Berlin: Springer-Verlag; 1991.

[63] Bolch BW. More on unbiased estimation of the standard deviation. The American Statistician. 1968;22(3):27.

